# Marginal donor kidney in a marginal recipient: Five year follow-up

**DOI:** 10.4103/0971-4065.65306

**Published:** 2010-04

**Authors:** M. M. Bahadur, P. Binnani, R. Gupta, S. Pattewar

**Affiliations:** Department of Nephrology, Jaslok Hospital and Research Centre, Mumbai, India

**Keywords:** Autosomal dominat polycystic kidney disease, cadaveric donor, marginal donor

## Abstract

The widening gap between demand and supply of organs became apparent as organ shortage became more severe. Organs previously considered unsuitable for transplantation are currently being used. Autosomal dominant polycystic kidney disease (ADPKD) is a hereditary disease characterized by slow progressive cystic changes and deterioration of renal function. We provide our experience with an ADPKD patient who received a kidney from 38-year-old deceased donor ADPKD-affected kidney for renal transplantation.

## Introduction

It is proven that long-term survival is markedly better among patients who receive a donor kidney compared with patients who remain on the waiting list for a kidney. However, there is a wide gap between the need for organs and their supply. One approach towards increasing donor kidney transplantation has been acceptance of “marginal” kidneys. This issue is increasingly being discussed in recent literature. ADPKD (Autosomal Dominant Polycystic Kidney Disease) is a hereditary disease characterized by slow progressive cystic changes and deterioration of renal function. We provide our experience with an ADPKD patient who received a 38-year-old deceased donor ADPKD-affected kidney for renal transplantation.

## Case Report

A 42-year-old male patient, a known case of chronic kidney disease with end stage renal failure secondary to ADPKD, was on maintenance hemodialysis since 1997. Since he did not have suitable donor in the family he was registered for cadaver transplant. He poorly tolerated hemodialysis sessions because of recurrent intradialytic hypotension and anginal chest pain during each HD session. He underwent coronary angiography in 2003, which confirmed triple vessel disease. He underwent CABG in 2003. In postoperative period, he had a stormy course, complicated with delayed sternal wound healing and prolonged ICU stay.

This patient showed no improvement in his functional status and also no symptomatic relief post CABG. He experienced anginal recurrences during dialysis sessions. He was the only earning member and had lot of family responsibilities. He was called in for cadaver renal transplant on 11/11/2004. The donor was a 38-year-old female, brain dead due to head injury, admitted at another hospital. At the time of organ recovery, both donor kidneys were studded with several cysts, 4 - 12 mm in size and were approximately 12 cm in longitudinal axis. The Serum creatinine concentration of the donor was 1.0 mg/dl.

After serious consideration, we decided to go ahead with the transplantation. The recipient was fully informed about the marginal status of the kidney before giving consent to transplantation. Surgery was uncomplicated and the kidney functioned immediately after transplant. The recipient was treated with cyclosporine, prednisone and azathioprine. Serum creatinine on day 20 was 1.4 mg/dL. The other kidney was also transplanted at the retrieving centre but unfortunately suffered graft loss.

Three months later, he had high-grade fever and right lower lobe pneumonitis. His Serum creatinine went up to 2.5mg/dL. Though he recovered from sepsis, his renal function remained same. Two years later, in 2006, in view of creeping creatinine and polycystic pathology, cyclosporine was switched to sirolimus, his Serum creatinine came down and stabilized to 1.8-2 mg/dL. He did not have any further episodes of infection also no cyst-related complications were found.

**Figure 1:**
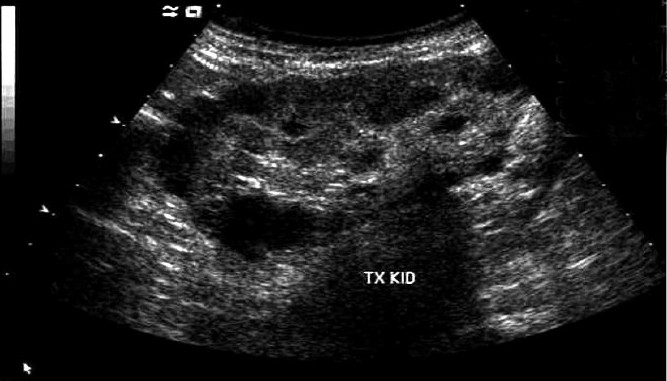
Recent ultrasonography of transplant kidney

Presently his renal function is stabilized with S. creatinine around 2.5 mg/dL. He experiences much less anginal episodes than earlier. Recent ultrasound of transplant kidney is shown in Figure 1. Kidney size is around 11 cm (approximately same as on the day of transplant) with multiple cysts, ranging in size from 5 to 25 mm. in diameter.

## Discussion

The 21^st^ century has come as an era of chronic diseases. Transplantation changed natural history and prognoses of some chronic kidney diseases. Widening gap between demand and supply of organs became apparent as organ shortage became more severe. Organs previously considered unsuitable for transplantation are currently being used. A new class of organ donor termed the “marginal” donor, also referred to as the “expanded” donor and “extended criteria” donor has emerged. Example of marginal donors includes non-heart-beating donors, donors with well-controlled hypertension, diabetes or mild proteinuria, donors older than 60 years and transplantation across the blood groups. We report our experience of adult cadaveric donor polycystic kidney.

There is approximately a 10-year lag period between the onset of symptoms and progression to end-stage renal failure when autosomal dominant polycystic kidney disease is considered. The chance of developing ESRD in ADPKD patients is 23% by 50 years and 48% by 73 years of age. As 85% of people with ADPKD are asymptomatic until the fourth decade of life, some may die of unrelated causes their organs might be offered for organ donation.[1]

Olsburgh *et al*. proposed some criteria for accepting such donors: donor less than 50 years of age, kidney size <15 cm in bipolar length, with normal Creatinine at the time of retrieval, pre-transplant renal biopsy is performed and results are discussed with pathologist, nephrologist and surgeon prior to considering transplantation, cold ischemia time is preferably <12 hours and not >24 hours. In these cases, polycystic kidneys can be accepted for transplantation in recipients, who may have a life expectancy of 10 years or less and who are fully informed regarding consent to receiving a polycystic graft.[2]

In the literature, there are around 12 successful cases of polycystic kidneys being used for transplantation. Many of these reports have follow-up ranging from 1 to 15 years.[3,4] Available data suggests that the expected graft survival of a normal or moderately enlarged polycystic kidney with normal function may be about 10 years.[5]

With improvement in short and long term graft and patient survival and quality of life after renal transplantation, there is justification for use of such kidneys when intervals between end stage renal disease and graft survival period are compared.[6]

In our patient, size of the transplant kidney did not change much from the day of transplant. This patient has been receiving Sirolimus for more than two years, which might have prevented cyst progression. In a two-year retrospective study in renal transplant recipients, Sirolimus was shown to reduce native kidney volume by 25%.[7]

## Conclusion

Cadaveric kidneys showing early signs of polycystic kidney disease with normal renal function should be considered acceptable for renal donation to recipients who may have a life expectancy of 10 years or less and who are fully informed regarding consent to receiving a polycystic graft. These organs provide the recipient a safe, reasonable period of graft survival and improve their quality of life.
